# A Stepwise Progression of Acute Bilateral Visual Loss Due to Onodi Cell Sinusitis

**DOI:** 10.7759/cureus.47359

**Published:** 2023-10-20

**Authors:** Hiroki Tamura, Takanori Uehara, Yu Li, Kazuki Yamasaki, Masatomi Ikusaka

**Affiliations:** 1 General Medicine, Chiba University Hospital, Chiba, JPN; 2 Otorhinolaryngology - Head and Neck Surgery, Chiba University Hospital, Chiba, JPN

**Keywords:** onodi cell, diagnostic delay, aspergillus fumigatus, acute visual loss, acute invasive fungal sinusitis

## Abstract

Acute visual loss in an immunocompromised patient may be caused by acute invasive fungal sinusitis (AIFS), even if symptoms include only mild headache and computed tomography (CT) shows only mild sinusitis, especially of the Onodi cell. Herein, we report a case of a 71-year-old man with a medical history of dermatomyositis and type 2 diabetes mellitus who presented with a stepwise progression of acute bilateral visual loss, mild headache, and altered consciousness. Initially, as the plain cranial CT showed only mild fluid retention in the posterior ethmoid sinus without bone destruction, the sinusitis was considered unrelated to the visual loss. Afterward, however, contrast-enhanced cranial magnetic resonance imaging (MRI) showed mucosal thickening, fluid retention in the posterior ethmoid sinus, and spread of the contrast medium over the dura around the right posterior ethmoid sinus and bilateral optic nerve tracts. *Aspergillus fumigatus* was identified from endoscopic drainage of the sinus. The patient was diagnosed with AIFS and treated with amphotericin B 350 mg/day. The altered sensorium and headache rapidly improved, and his left visual acuity improved to counting fingers. Although AIFS is rare, it can cause severe sequela or death due to vascular or direct intracranial invasion. Therefore, immediate drainage of the sinus and intravenous antifungal therapy are essential for AIFS. Our findings will help physicians make accurate and rapid diagnoses of AIFS in future cases.

## Introduction

Acute invasive fungal sinusitis (AIFS) is rare but can be fatal due to vascular or direct intracranial invasion [1]. Aspergillus spp. are the most common pathogens in AIFS [2]. Diabetes mellitus (DM) and the use of immunosuppressive drugs are well-known risk factors [1]. Sinusitis is a high-prevalence disease experienced by 12% of the population [3]; meanwhile, AIFS is very rare, with a weighted estimate of only 340 cases in the United States in 2018 [4]. Although AIFS often causes decreased visual acuity with a rapid time course of days to a few weeks, the pain can be mild. The Onodi cell, an anatomic variant of the ethmoid sinus, is intimately related to the optic nerve tracts, and the optic nerve is vulnerable to inflammation of the Onodi cell [5]. The diagnosis of AIFS could be challenging because of its rarity and the pain is only mild. Herein, we report a case of a 71-year-old immunocompromised man with a stepwise progression of acute bilateral visual loss, mild headache, and altered consciousness. Our findings will help physicians make accurate and rapid diagnoses in future cases to prevent severe sequela or death by AIFS.

## Case presentation

**Figure 1:**
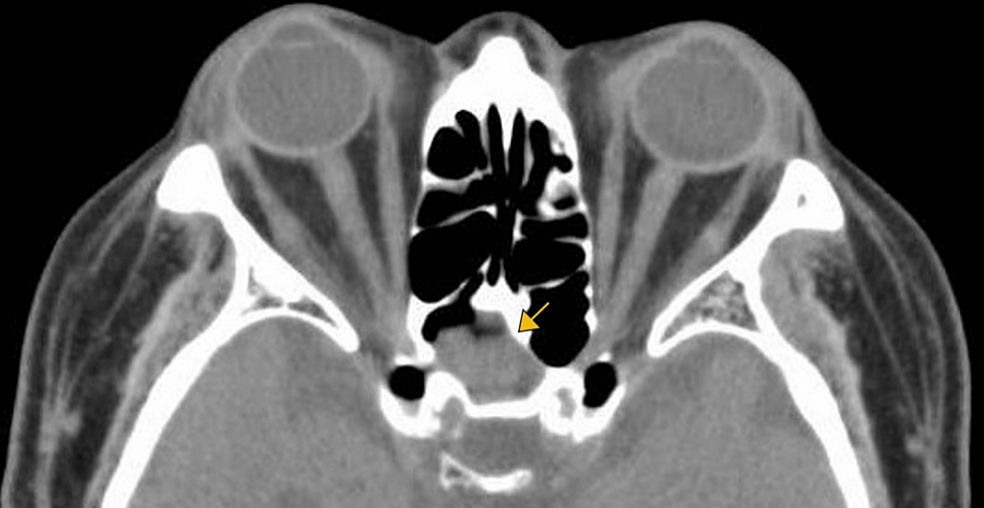
Computed tomography shows mild accumulation of fluid in the right posterior ethmoid sinus without bone destruction (arrow)

A 71-year-old man with a medical history of dermatomyositis and type 2 DM, for which he was being given prednisolone 15 mg/day, tacrolimus 1.5 mg/day, and a combination of oral hypoglycemic agents (teneligliptin, pioglitazone, miglitol, and glimepiride), presented with a stepwise progression of acute bilateral visual loss and mild headache. Headache and right-sided blurred vision without orbital pain started eight weeks before the patient presented to our clinic; furthermore, the patient’s visual acuity had deteriorated to light perception seven weeks before presenting. At that time, only simple diabetic retinopathy was observed on fundus examination. Since cranial computed tomography (CT) showed only mild fluid retention in the posterior ethmoid sinus without bone destruction (Figure 1), the sinusitis was considered unrelated to the visual loss. The provisional diagnosis was ischemic optic neuropathy and bacterial posterior ethmoid sinusitis, which was considered to be unrelated to the visual loss. For six weeks, the patient’s general condition and visual loss remained unchanged. However, four days before presenting to our clinic, contralateral visual loss occurred, and somnolence worsened over the next three days. No nasal symptoms such as nasal obstruction, rhinorrhea, or posterior rhinorrhea were observed during the course of the illness.

**Figure 2:**
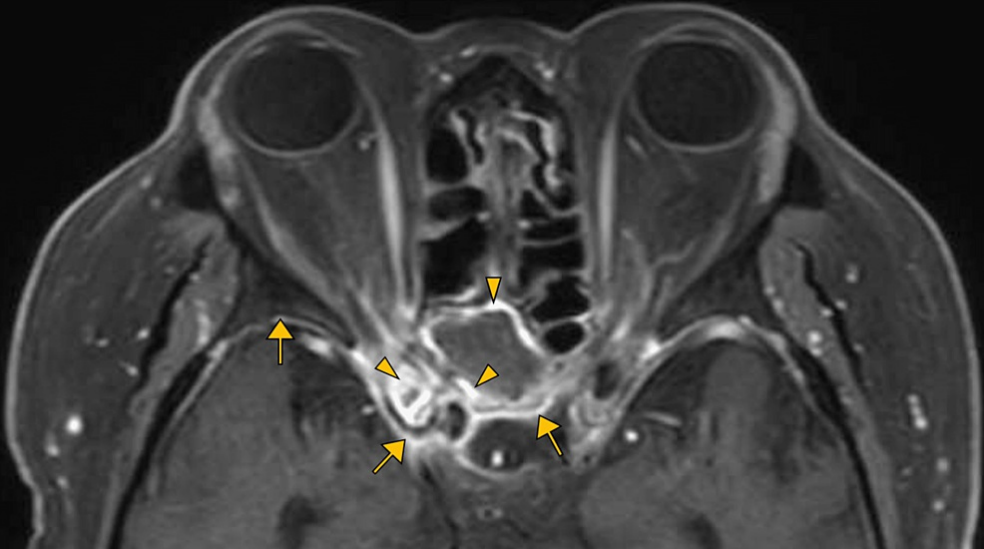
Contrast-enhanced magnetic resonance imaging (fat-suppressed T1-weighted image) shows mucosal thickening (arrowheads) and fluid retention in the right posterior ethmoid sinus and spread of the contrast medium over the dura around the right posterior ethmoid sinus (arrows) and bilateral optic nerve tracts (right predominant)

A physical examination at our clinic revealed mild altered sensorium (Glasgow coma scale: E3V4M6); the bilateral visual acuity was light perception, and the bilateral light reflex was negative. The patient had no neurological abnormalities other than impaired visual acuity, light reflex, and consciousness. Laboratory studies revealed a serum C-reactive protein level of 0.66 mg/dL and a hemoglobin A1c level of 11.2%. Contrast-enhanced cranial MRI revealed an anatomic variant of the posterior ethmoid sinus called the Onodi cell, with mucosal thickening and fluid retention, and the contrast medium had spread over the dura around the right posterior ethmoid sinus and bilateral optic nerve tracts (Figure 2). Cerebrospinal fluid analysis showed an initial pressure of 85 mmH_2_O, leukocyte count of 1 cell/3 μL, protein level of 216 mg/dL, and glucose level of 194 mg/dL (blood glucose level, 254 mg/dL). Cerebrospinal fluid Gram stain, acid-fast bacilli stain, and culture were negative. An otolaryngologist performed endoscopic drainage of the sinus, and *Aspergillus fumigatus* was identified. The patient was diagnosed with AIFS and treated with amphotericin B 350 mg/day. The sensorium and headache rapidly improved, and his left visual acuity improved to counting fingers.

## Discussion

The patient had AIFS due to Aspergillus. Fungal sinusitis is characterized by invasive (classified into acute invasive, chronic invasive, and granulomatous forms) and noninvasive (classified into allergic fungal sinusitis and mycetoma) forms [6]. Aspergillus spp. are the most common pathogens in AIFS, and together with Mucor spp. account for 90% of cases of AIFS [2]. AIFS is rare but can be fatal owing to vascular or direct intracranial invasion [1], especially when it occurs in cases of sinusitis associated with Onodi cells, as in our case. The patient's posterior ethmoid sinus was located laterally and superiorly to the sphenoid sinus (Figure 2). This anatomic variant of the ethmoid sinus has been reported in 7%-65% of people and is called the Onodi cell, which is intimately related to the optic nerve tracts [5]. Therefore, the optic nerve is more vulnerable to inflammation of the Onodi cell [5]. Patients with AIFS with orbital involvement had a significantly higher incidence of sinus involvement with the ethmoid (100%) and sphenoid sinus (88.9%), DM complications (100%), and decreased visual acuity (88.9%) than those with orbital sparing [7]. Our patient was being given prednisolone and had a hemoglobin A1c level of 11.2%, notably, the use of immunosuppressive drugs and poorly controlled DM are well-known risk factors. Among patients with AIFS, 36.8% and 15.7% have a history of corticosteroid exposure and DM (poorly controlled DM accounts for 10.5%), respectively [1]. Furthermore, in AIFS, the sensitivity to pain includes orbital pain (66.7%), headache (44.4%), and cheek pain (44.4%); therefore, painless AIFS is not uncommon. Moreover, the pain of AIFS could be mild [7]. The mortality rate of AIFS is 50%-80%, owing to difficulties in early diagnosis [2]. Treatment of AIFS includes drainage combined with systemic antifungal therapy [8]. In our case, a diagnostic delay occurred because the headache was mild and the CT showed only mild sinusitis. Differential diagnoses include ischemic optic neuropathy and bacterial sinusitis. Regarding pain, both ischemic optic neuropathy and AIFS can be painless or present only with mild pain. Differentiating the cause of decreased visual acuity, whether bacterial or fungal sinusitis, by considering the patient’s history is difficult [9]. In cases of acute visual loss in an immunocompromised patient, it is essential to consider the differential diagnosis of potentially life-threatening AIFS, even if the symptoms or imaging findings are mild.

## Conclusions

AIFS is a fatal disease that can cause severe visual deficits even when the life is saved, as in our case. AIFS is typically rapidly progressive within a few days and weeks; however, when diagnostic delays occur, a stepwise progression of deterioration occurs, as in our case. Therefore, although rare, AIFS should be included in the differential diagnosis of acute or subacute visual loss in immunocompromised patients, particularly those with Onodi cells, to avoid a diagnostic delay, even if symptoms include only mild headache and CT shows only mild sinusitis. Immediate drainage of the sinus and intravenous antifungal therapy are essential in preventing serious sequelae and death.
